# Supplementary material for: Effect of basal luteinizing hormone/follicle-stimulating hormone ratio on clinical outcome of In Vitro fertilization in patients with polycystic ovarian syndrome: a retrospective cohort study
Source: PeerJ. 2024 Nov 26;12:e18635. doi: 10.7717/peerj.18635 (PMC11606322; doi:10.7717/peerj.18635)
Supplement: Supplemental Information 2 [file peerj-12-18635-s002.docx]

**Supplementary Materials for** **Effect of Basal Luteinizing Hormone/Follicle-Stimulating Hormone Ratio on Clinical Outcome of In Vitro Fertilization in Patients with Polycystic Ovarian Syndrome: A Retrospective Cohort Study**

**Supplemental Table S1: Correlation between LH/FSH ratio and other indicators between groups of different LH/FSH ratio**

|  | LH/FSH ratio (Gonadotropin agonist protocol)  β | LH/FSH ratio (Mild stimulation protocol)  β |
| --- | --- | --- |
| Age (years old) | -0.048 | -0.107 |
| BMI (kg/m^2^) | -0.092* | -0.158 |
| Infertility duration (years) | 0.010 | -0.083 |
| FSH (IU/L) | 0.020 | 0.061 |
| LH (IU/L) | 0.905** | 0.896** |
| Estradiol (ng/ml) | 0.224** | 0.285* |
| Testosterone (ng/ml) | 0.204** | 0.276* |
| Total gonadotropin dose (IU) | -0.053 | -0.077 |
| Total gonadotropin time (days) | 0.092* | -0.186 |
| Estradiol level on HCG trigger day (ng/ml) | 0.014 | 0.090 |
| Endometrial thickness on HCG trigger day (mm) | -0.017 | 0.021 |
| Number of oocytes retrieved | 0.011 | 0.148 |
| Number of embryos transferred | -0.089* | -0.238* |

*P < 0.05，**P < 0.01

**Supplemental Table S2: Correlation between LH/FSH ratio and other indicators between groups of different ovulation stimulation protocols when LH/FSH ratio ≥2**

|  | LH/FSH ratio (Gonadotropin agonist protocol)  β |
| --- | --- |
| Age (years old) | -0.110 |
| BMI (kg/m^2^) | -0.059 |
| Infertility duration (years) | -0.190* |
| FSH (IU/L) | -0.081 |
| LH (IU/L) | 0.627** |
| Estradiol (ng/ml) | 0.182 |
| Testosterone (ng/ml) | 0.220* |
| Total gonadotropin dose (IU) | -0.116 |
| Total gonadotropin time (days) | 0.033 |
| Estradiol level on HCG trigger day (ng/ml) | -0.027 |
| Endometrial thickness on HCG trigger day (mm) | -0.087 |
| Number of oocytes retrieved | 0.081 |
| Number of embryos transferred | -0.028 |

*P < 0.05，**P < 0.01

**Supplemental Table S3: Adjusted live birth between groups of different ovulation stimulation protocols when LH/FSH ratio ≥2**

|  | OR | 95% CI | P |
| --- | --- | --- | --- |
| LH/FSH | 0.687 | 0.373, 1.268 | 0.230 |

OR: odds ratio; CI: confidence interval
